# The Contribution of Insomnia and Obstructive Sleep Apnea on the Transition from Acute to Chronic Painful Temporomandibular Disorders and their Persistence: A Prospective 3-Month Cohort Study

**DOI:** 10.1080/24740527.2023.2266738

**Published:** 2023-11-28

**Authors:** Sherif M. Elsaraj, Mervyn Gornitsky, Richard Hovey, Firoozeh Samim, Zovinar Der Khatchadourian, Ana Velly

**Affiliations:** a Faculty of Dental Medicine and Oral Health Sciences, McGill University, Montreal, Quebec, Canada; b Department of Dentistry, Jewish General Hospital, Montreal, Quebec, Canada

**Keywords:** temporomandibular disorders, chronic pain, acute pain, transition, cohort study, risk factors, insomnia, obstructive sleep apnea, disability, dysfunction

## Abstract

**Background:**

Insomnia, and Excessive Daytime Sleepiness (EDS), a surrogate marker of Obstructive Sleep Apnea (OSA), are common sleep-related conditions among painful temporomandibular disorders (TMD) patients. OSA was found to increase the risk of chronic painful TMD.

**Aims:**

This prospective cohort study aims to determine the contribution of insomnia and EDS on acute to chronic painful TMD transition as well as its persistence when chronic pain is defined by: (i) duration (> 3 months), and (ii) dysfunction (Graded Chronic Pain Scale [GCPS II-IV]).

**Methods:**

From 456 patients recruited between 2015 to 2021, through four locations in Canada, 378 completed the follow-up. A diagnosis was obtained using the Research Diagnostic Criteria or the Diagnostic Criteria for TMD. Insomnia was assessed with the Insomnia Severity Scale (ISS), and OSA was assessed using the Epworth Sleepiness Scale (ESS) which measures EDS, both at baseline. Patients completed the GCPS form at baseline and 3-month follow-up.

**Results:**

Borderline associations were found between EDS and the transition or persistence of chronic painful TMD when chronic pain was defined by pain duration (RR _adjusted_duration_ = 1.11, *P* = 0.07) and dysfunction (RR_adjusted_dysfunction_ =1.40, *P* = 0.051). Furthermore, EDS was specifically associated with persistent painful TMD when chronic pain was defined by pain duration (RR = 1.13, 95%CI: 1.00-1.26, P = 0.04). Insomnia was not related to the study outcomes (RR_adjusted_duration_ = 0.94, *P* = 0.27, RR_adjusted_dysfunction_ =1.00, *P* = 0.99).

**Conclusion:**

Results indicate that EDS contrary to insomnia predicted the persistence of chronic painful TMD at a 3-month follow-up.

## RÉSUMÉ

**Contexte:** L’insomnie et la somnolence excessive en journée, un marqueur substitut de l’apnée du sommeil obstructive, sont des affections courantes liées au sommeil chez les patients souffrant de troubles temporo-mandibulaires douloureux. On a découvert que l’apnée obstructive du sommeil augmentait le risque de troubles temporo-mandibulaires douloureux chroniques.

**Objectifs:** Cette étude de cohorte prospective vise à déterminer la contribution de l’insomnie et de la somnolence excessive en journée à la transition des troubles temporo-mandibulaires douloureux aigus à chroniques, ainsi qu’à leur persistance lorsque la douleur chronique est définie par : (i) la durée (> 3 mois), et (ii) la dysfonction (Échelle de douleur chronique graduée [GCPS II-IV]).

**Méthodes:** Sur 456 patients recrutés entre 2015 et 2021 dans quatre endroits au Canada, 378 ont terminé le suivi. Un diagnostic a été établi en utilisant les Critères de diagnostic pour la recherche ou les Critères de diagnostic pour les troubles temporo-mandibulaires douloureux. L’insomnie a été évaluée à l’aide de l’Indice de sévérité de l’insomnie (ISI) et l’apnée obstructive du sommeil a été évaluée à l’aide de l’Échelle de somnolence d’Epworth (ESS), qui mesure la somnolence excessive en journée, tous deux au début de l’étude. Les patients ont rempli le formulaire GCPS au début de l’étude et lors du suivi à trois mois.

**Résultats:** Des associations marginales ont été trouvées entre la somnolence excessive en journée et la transition ou la persistance des troubles temporo-mandibulaires douloureux chroniques lorsque la douleur chronique était définie par la durée de la douleur (RR ajusté pour la durée = 1,11, P = 0,07) et la dysfonction (RR ajusté pour la dysfonction = 1,40, P = 0,051). De plus, la somnolence excessive en journée était spécifiquement associée à la persistance des troubles temporo-mandibulaires douloureux lorsque la douleur chronique était définie par la durée de la douleur (RR = 1,13, IC à 95 % : 1,00-1,26, P = 0,04). L’insomnie n’était pas liée aux résultats de l’étude (RR ajusté pour la durée = 0,94, P = 0,27, RR ajusté pour la dysfonction = 1,00, P = 0,99).

**Conclusion:** Les résultats indiquent que la somnolence excessive en journée, contrairement à l’insomnie, prédisait la persistance des troubles temporo-mandibulaires douloureux chroniques lors du suivi de trois mois.

## Introduction

Temporomandibular disorders (TMDs) are a group of musculoskeletal conditions characterized by pain and dysfunction in the muscles of mastication, the temporomandibular joints, or both.^1,2^ TMD is the second most commonly occurring musculoskeletal disorder after chronic lower back pain^2^ and is an important public health concern because it affects a significant portion of the general population, with prevalence estimates ranging from 5% to 12%.^3,4^ Many factors have been identified to increase the risk of TMD.^5–9^

Insomnia is common among individuals with TMD (36%).^10^ Insomnia is a sleep disorder that is defined as the subjective experience of difficulty initiating sleep, maintaining sleep, and/or early morning awakening for at least 3 nights a week for three consecutive months, while there is adequate opportunity for undisturbed sleep and the reports are also not adequately explained by other mental or physical health issues. To date, there is no existing evidence that insomnia increases the risk of TMD.

Obstructive sleep apnea (OSA) is another breathing disorder that is a serious and potentially life-threatening condition characterized by brief interruptions of breathing during sleep.^10^ The OPPERA (Orofacial Pain: Prospective Evaluation and Risk Assessment) case–control study found that patients with TMD were almost four times more likely to present with OSA compared to controls (odds ratio = 3.63; 95% confidence interval [CI] 2.03–6.52).^11^ Moreover, the prospective cohort of the OPPERA study found that OSA increased the risk of TMD onset (adjusted hazard ratio = 1.73; 95% CI 1.14–2.62). The OPPERA study used some questions from the Pittsburgh Sleep Quality Index and the STOP-BANG questionnaire.^11^

Although the relationship between OSA and painful TMD risk has been demonstrated, it is not yet clear whether sleep disorders are associated with the perpetuation of painful TMD.

Thus, the primary aims of this study were to determine whether insomnia, and excessive daytime sleepiness, a consequence of OSA (EDS), were associated with the transition from acute to chronic painful TMD, as well as its persistence. In this study, chronic painful TMD was defined as (1) recurrent or persistent painful TMD for more than 3 months of follow-up and (2) dysfunction as classified by the Graded Chronic Pain Scale (GCPS) grades II–IV.^12^

The rationale to define chronic pain outcomes using pain duration or dysfunction follows the recommendation of the International Association for the Study of Pain, which defines chronic pain as recurrent or persistent pain lasting for more than 3 months with significant emotional distress and/or functional disability.^13^ Our choice to categorize dysfunction using the GCPS (grades II–IV) was influenced by previous studies.^14–16^ The study hypotheses are that insomnia and EDS are associated with the transition from acute to chronic painful TMD as well as its persistence. To date, we are not aware of any study investigating whether insomnia and EDS are associated with the transition from acute to chronic painful TMD as well as its persistence.

## Materials and Methods

### Study Design and Study Population

This prospective cohort study is part of a global project called ACTION (Acute to Chronic Pain Transition), aimed at identifying predictive factors contributing to the transition from acute to chronic painful TMD and its persistence. The ACTION program received approvals from the McGill Institutional Review Board in Montreal, Canada (Approval No. A12-M113-14A) and the Dental Specialists Group in Ottawa, Ontario (Approval No. 240–400).

Eligible patients with acute or chronic painful TMD were recruited from four different sites between August 2015 and March 2021. These locations were the Jewish General Hospital general dental clinic, the Faculty of Dentistry of McGill University oral diagnosis clinic, the Montreal General Hospital dental department, and the Dental Specialists Group TMD-specialized clinic. To be included in this study, the study required patients to be 18 to 85 years of age with a positive diagnosis of painful TMD (muscle and/or joint) in accordance with research diagnostic criteria^17^ or the diagnostic criteria for TMDs.^18^ The exclusion criteria were patients who had other orofacial pain (e.g., dental pain), cancer, no access to a telephone and those who were unable to provide informed consent or were incapable of understanding French or English. Participants at the Jewish General Hospital were recruited by M.G. and S.M.E., Montreal General Hospital by F.S., the oral diagnosis clinic by Z.K., and the Dental Specialists Group by S.M.E. These are trained dentists in the field of TMD and orofacial pain.

### Acute and Chronic Painful TMD Cohorts and Outcomes

At baseline (first visit), pain duration was assessed by asking patients, “How many years or months ago did the pain in the jaw, temple, in the ear, or in front of the ear first begin?” Patients reporting painful TMD for 3 months or less were included in the acute cohort and those reporting pain for more than 3 months were enrolled in the chronic cohort.

The primary study outcomes were the transition from acute to chronic painful TMD and its persistence. Two criteria were used to define chronic painful TMD: (1) pain lasting for more than 3 months (no versus yes) and (2) dysfunction (no [GCPS I] and yes [GCPS II–IV]).^12^ The GCPS grades are low-intensity pain, no disability (grade I); high-intensity pain, without pain-related disability (grade IIa); high-intensity pain, with low pain-related disability (grade IIb), moderately limiting (grade III), and severely limiting (grade IV). The GCPS scoring is based on the subject’s responses to several items: (1) current, (2) worst, and (3) average pain intensity (0–10 numeric scales) and (4) pain-related disability days and pain-related interference with daily activities, work, and social or family activities (0–10 numeric scales). Characteristic pain intensity (CPI) measured by the GCPS is the average of 0 to 10 ratings of current, worst, and average pain in the previous 3 months multiplied by 10. The disability score is the average of three 0 to 10 interference ratings: daily activities, work, and social or family activities multiplied by 10 in the previous 3 months. The secondary outcomes were also the transition and persistence states, both stratified by duration or dysfunction.

### Assessment of the Potential Predictive Factors

#### Insomnia Screening Instrument

A seven-item validated questionnaire called the Insomnia Severity Index,^19^ with excellent sensitivity (94%) and specificity (94%), was used to evaluate sleep disturbances.^20^ This instrument evaluates (1) the severity of sleep onset, (2) sleep maintenance, (3) early morning awakening problems, (4) satisfaction with current sleep pattern, (5) sleep-related interference with daily function, (6) noticeability of impairment attributed to sleep problems, and (7) level of distress caused by sleep problems. Participants rate each of these factors on a 5-point (0–4) scale, with possible scores ranging from 0 (*no clinically significant insomnia*) to 28 (*severe clinical insomnia*). A total score is calculated, and sums between 0 and 7 indicate no clinically significant insomnia, sums between 8 and 14 indicate subthreshold insomnia, and a score of 15 or more represents clinically significant insomnia. The scoring cutoffs are less than 14: no or subthreshold insomnia and greater than or equal to 15: clinically significant insomnia.

#### EDS Screening Instrument

Comprehensive sleep evaluation for the likelihood of having excessive daytime sleepiness, a surrogate marker for OSA, was assessed using the validated Epworth Sleepiness Scale (ESS) questionnaire.^21^ This 8-item validated questionnaire is used to assess EDS in patients who are at high risk for OSA. The score for the ESS is the sum of the score of all questions. A score between 0 and 9 is considered normal, whereas scores between 10 and 24 indicate that expert medical advice is required. For instance, scores of 11 to 15 are shown to indicate the possibility of mild to moderate OSA, whereas scores of 16 and above indicate the possibility of severe OSA. The ESS instrument is widely used in sleep clinics to screen for suspected OSA before authorizing a polysomnography study.^22^ The ESS has excellent sensitivity (93.5%) and specificity (100%).^20^

In the literature, we noted inconsistencies in the reporting of sensitivity and specificity of the ESS instrument for OSA screening (EDS). For instance, Sommer et al.^20^ showed that ESS has an excellent sensitivity (93.5%) and specificity (100%) to screen for OSA using a cutoff >10. However, the Vana et al. study,^22^ using the same parameters, showed that ESS has sensitivity of 31.3% and specificity of 53.3%. This is a very low specificity and sensitivity and was obtained using ESS score >10 to determine its predictive ability for OSA by comparing it to their polysomnographic testing results.

### Assessment of Putative Confounders and Effect Modifiers

Regarding the outcome evaluated in terms of pain duration, potential confounders and effect modifiers encompassed acute and chronic pain status, CPI, psychological aspects (anxiety, depression), gender, and age. For the outcome centered around dysfunction, conceivable covariates included acute and chronic pain conditions, dysfunction, psychological aspects (anxiety, depression), gender, and age. It was not necessary to add CPI in the dysfunctional model because CPI is part of the equation to calculate the GCPS score.^12^

Screening for psychological factors: anxiety and depression was accomplished with the Patient Health Questionnaire-4.^23^ The scoring cutoffs for the Patient Health Questionnaire-4 are as follows: <3: no anxiety or depression and ≥3: anxiety or depression.

### Statistical Analysis

Chi-square, Fisher’s exact test, analysis of variance, and Student’s *t* test were used to test statistical differences between categories of painful TMD groups relative to insomnia and EDS, acute and chronic pain status, GCPS grades (GCPS I–IV), CPI, age, sex, and psychological factors.

Prior to the start of the study, a sample size calculation was performed. Because there are no data in the literature on painful TMD for expected outcomes and frequencies of the potential risk factors among those with and without the study outcomes, we performed the sample size estimation using the percentage of outcomes among those not exposed ranging from 30% to 50% and risk ratio (RR) equal to 2. Based on these values, we would need 42 patients exposed and 42 not exposed to be able to reject the null hypothesis that this Odds Ratio (OR) is equal to 1 with a probability (power) of 80%. The type I error probability associated with this test of this null hypothesis is 0.05. The dropout-adjusted sample size was 105 (total sample size = 84/(1, anticipated dropout rate 20%)).

#### Primary Analysis

Binary logistic regression analyses (PROC GENMOD, SAS ^24^) were conducted to determine whether insomnia and EDS were associated with the transition from acute to chronic painful TMD, as well as its persistence. For aim 1, the dependent variable was the presence or absence of chronic painful TMD at 3-month follow-up (no: CPI = 0; yes: CPI > 0). For aim 2, the dependent variable was presence (GCPS II–IV) or absence (GCPS I) of dysfunction at 3-month follow-up. The independent variables were the potential predictive factors under investigation: insomnia and EDS. The putative confounders and effect modifiers were listed above.

The RR and their 95% CIs were estimated in all analyses. We employed three commonly used analyses: (1) crude analyses, (2) multivariable model including all potential predictors and confounders, and (3) a parsimonious multivariable model that incorporated variables associated with the outcome, confounders (both historical and identified in the analyses), and variables that improved the precision of the model. The likelihood ratio test was used to assess the significance of the RR and interactions in the model. All analyses were performed using the statistical software package SAS, with the significance level for type I error set at the 0.05 level.

#### Secondary Analysis

Interaction terms between each sleep disorder and acute–chronic pain status and GCPS status at baseline were introduced. This was done to determine whether the sleep disorder risks were modified by these covariates. The interaction terms were retained in the model only if the significance level of the regression coefficient was ≤0.10. Furthermore, the logistic regression analyses were stratified by pain duration (acute [≤3 months], chronic [>3 months]) and dysfunction (no [GCPS I] and yes [GCPS II–IV]). Finally, logistic regression analyses were performed to evaluate the association between insomnia and EDS scores and the painful TMD outcomes.

## Results

### Transition and Persistence of Chronic Painful TMD Cohorts Defined by Pain Duration

Of a total of 516 patients informed of the study, 10 refused to participate and 50 were not eligible due to lack of time and distress. Table 1 shows the characteristics of acute and chronic painful TMD cohorts at the first visit (baseline). The total number of patients with painful TMD enrolled was 456, of which 123 patients were included in the acute cohort (≤3 months; 26.96%) and 333 in the chronic cohort (>3 months; 73.03%). Insomnia and EDS were slightly more common in the chronic painful TMD cohort compared to the acute cohort (insomnia: 28.23% versus 22.76%, *P* = 0.24; EDS: 49.70% versus 44.72%, *P* = 0.34). No statistically significant differences were found between the groups. There were more females in the chronic painful TMD cohort compared to the acute cohort (78.68% versus 69.92%, *P* = 0.05).

**Table 1.** Baseline characteristics of acute and chronic TMD-related pain cohorts defined by duration.

Risk factors and covariates	Category	Acute cohort (≤3 months)	Chronic cohort (>3 months)	*P* value
		123 (26.96%)	333 (73.03%)	
Insomnia, *n* (%)	>14	28 (22.76)	94 (28.23)	0.24
	≤14	95 (77.24)	239 (71.77)	
EDS, *n* (%)	>5	55 (44.72)	164 (49.70)	0.34
	≤5	68 (55.28)	166 (50.30)	
Sex, *n* (%)	Female	86 (69.92)	262 (78.68)	0.05
	Male	37 (30.08)	71 (21.32)	
Psychological factors, *n* (%)^a^	≥3	69 (56.10)	207 (62.16)	0.24
	<3	54 (43.90)	126 (37.84)	
Age (years)	Mean	42.64	41.74	0.60
	Median	39.00	39.00	
	SD	16.11	16.29	
CPI (0–100 NRS)	Mean	57.43	57.41	0.98
	Median	56.66	60.00	
	SD	21.42	21.65	

^a^Psychological factors: ≥3 = yes, <3 = no.

NRS = numeric rating scale.

Table 2 shows the baseline profile of the acute and chronic painful TMD cohorts that completed the 3-month follow-up. The number of participants who completed the follow-up was 378 out of the total enrolled of 456 (82.89%). Insomnia and EDS remained slightly higher in the chronic pain cohort compared to the acute cohort (insomnia: 30.80% versus 23.53%, *P* = 0.17; EDS: 49.08% versus 45.10%, *P* = 0.49). No statistically significant differences were found between the groups.

**Table 2.** Baseline profile of the acute and chronic TMD-related pain cohorts who completed the 3-month follow-up.

Risk factors and covariates	Category	Acute cohort (≤3 months)	Chronic cohort (>3 months)	Total	*P* value
		102 (26.98%)	276 (73.02%)	378	
Insomnia, *n* (%)^a^	>14	24 (23.53)	85 (30.80)	109 (28.84)	0.17
	≤14	78 (76.47)	191 (69.20)	269 (71.16)	
EDS, *n* (%)^b^	>5	46 (45.10)	134 (49.08)	180 (48.00)	0.49
	≤5	56 (54.90)	139 (50.92)	195 (52.00)	
Sex, *n* (%)	Female	72 (70.59)	214 (77.54)	286 (75.66)	0.16
	Male	30 (29.41)	62 (22.46)	92 (24.34)	
Psychological factors, *n* (%)^c^	≥3	58 (56.86)	176 (63.77)	234 (61.90)	0.22
	<3	44 (43.14)	100 (36.23)	144 (38.10)	
Age (years)	Mean	42.34	41.52	41.59	0.74
	Median	39.00	39.00	39.00	
	SD	16.70	16.15	16.14	
CPI (0–100 NRS)	Mean	57.94	57.29	57.46	0.71
	Median	60.00	60.0	60.0	
	SD	20.26	21.73	21.33	

^a^Insomnia: ≤14 = no, >14 = yes.

^b^EDS: ≤5 = no, >5 = yes.

^c^Psychological factors: ≥3 = yes, <3 = no.

NRS = numeric rating scale.

From the acute painful TMD cohort (pain duration ≤3 months since first visit) including 102 patients who completed the 3-month follow-up, half of the patients presented a transition to chronic painful TMD (*n* = 52), and the other half had no pain (*n* = 50). From the chronic painful TMD cohort (pain duration >3 months since first visit), 75.72% had persistent chronic pain (*n* = 209/276), whereas almost a quarter of patients had no pain at 3-month follow-up (*n* = 67).

Table 3 shows the baseline profile of patients with and without chronic painful TMD at 3-month follow-up (261 versus 117). Almost one-third of those with (29.50%) and without (27.35%) chronic painful TMD had insomnia (*P* = 0.67). EDS was more common among the patients with chronic painful TMD (50.58%) than those without pain (42.24%). However, this difference was not statistically significant (*P* = 0.13).

**Table 3.** Baseline profile of patients with chronic TMD-related pain and those without pain at 3-month follow-up.

Risk factors and covariates	Category	No TMD-related pain	Chronic TMD-related pain	*P* value
		117 (30.95)	261 (69.05)	
Insomnia, *n* (%)^a^	>14	32 (27.35)	77 (29.50)	0.67
	≤14	85 (72.65)	184 (70.50)	
EDS, *n* (%)^b^	>5	49 (42.24)	131 (50.58)	0.13
	≤5	67 (57.76)	128 (49.42)	
Sex, *n* (%)	Female	76 (64.96)	210 (80.46)	0.001
	Male	41 (35.04)	51 (19.54)	
Psychological factors, *n* (%)^c^	≥3	69 (58.97)	165 (63.22)	0.43
	<3	48 (41.03)	96 (36.78)	
Mean age (95% CI)	Years	40.11 (37.17–43.06)	42.39 (40.42–44.36)	0.21
Mean CPI (95% CI)	0–100 NRS	51.06 (47.24–54.88)	60.41 (57.85–62.96)	<0.0001

^a^Insomnia: ≤14 = no, >14 = yes.

^b^EDS: ≤5 = no, >5 = yes.

^c^Psychological factors: ≥3 = yes, <3 = no.

NRS = numeric rating scale.

#### The Association between Sleep Disorders and the Transition or Persistence of Chronic Painful TMD

Table 4 highlights EDS and insomnia crude and multivariable logistic regression analyses. RR estimates showed no significant associations between insomnia and the transition or persistence risk at 3-month follow-up (RR_crude_ = 1.03, RR_modelII_ = 0.94, *P* = 0.27). A borderline association was found between the transition or persistent risk and EDS (RR_ModelIII_ = 1.11, *P* = 0.07) when the model included the acute and chronic pain status at baseline, CPI, sex, age, and psychological factors. To improve the precision of the model, insomnia was not included in model III.

**Table 4.** Crude and multivariable logistic regression analyses assessing the contribution of insomnia and EDS on transition or persistent TMD-related pain at 3-month follow-up.

Risk factors and covariates at baseline	Category	Model I RR (95% CI)	*P* value	Model II RR (95% CI)	*P* value	Model III RR (95% CI)	*P* value
Insomnia	≤14	1.0 (reference)	0.66	1.0 (reference)	0.27	Not in the model	
	>14	1.03 (0.89–1.19)		0.94 (0.84–1.05)			
EDS	≤5	1.0 (reference)	0.13	1.0 (reference)	0.19	1.0 (reference)	0.07
	>5	1.11 (0.97–1.27)		1.07 (0.97–1.18)		1.11 (0.99–1.25)	
Acute and chronic TMD-related pain	≤3 months	1.0 (reference)	0.0001	1.0 (reference)	0.002	1.0 (reference)	<0.0001
	>3 months	1.48 (1.21–1.81)		1.23 (1.08–1.40)		1.47 (1.20–1.79)	
Mean CPI	0–100 NRS	1.01 (1.00–1.01)	0.0001	1.00 (1.00–1.01)	0.008	1.00 (1.00–1.01)	0.0001
Sex	Male	1.0 (reference)	0.005	1.0 (reference)	0.07	1.00 (reference)	0.02
	Female	1.32 (1.09–1.61)		1.13 (0.99–1.29)		1.25 (1.04–1.51)	
Mean age	Years	1.00 (0.99–1.01)	0.21	1.00 (0.99–1.00)	0.70	1.00 (0.99–1.00)	0.54
Psychological factors^a^	<3	1.0 (reference)	0.44	1.0 (reference)	0.91	1.0 (reference)	0.35
	≥3	1.06 (0.92–1.22)		0.99 (0.89–1.11)		0.94 (0.83–1.07)	

Model I = crude analysis.

Model II = multivariable model including sleep disorders and all covariates: AIC: 446.83, AICC = 447.23, BIC = 478.20.

Model III = multivariable model including ESA/OSA and all covariates: AIC: 432.79, AICC = 433.10, BIC. = 460.24.

^a^Psychological factors ≥3 = yes, <3 = no.

NRS = numeric rating scale.

#### Secondary Analyses

##### EDS and Insomnia Binary Variables

No interactions were found between each sleep disorder and the acute and chronic status at baseline (*P* > 0.28). The stratified analysis found that insomnia (RR = 1.20, 95% CI 0.68–2.12, *P* = 0.52) and EDS (RR = 0.85, 95% CI 0.55–1.31, *P* = 0.45) were not related to the transition risk. However, a significant association was found between persistence of painful TMD and (RR_EDS_ = 1.13, 95% CI 1.00–1.26, *P* = 0.04). No association was found with insomnia (RR = 0.92, 95% CI 0.81–1.05, *P* = 0.22). These models included sex, age, psychological factors, and CPI.

##### EDS and Insomnia Scores

Regarding the score analysis, EDS score was not related to the transition or persistent risk (RR = 1.01, 95% CI 0.99–1.02, *P* = 0.32). The stratified analysis, however, found that EDS score contributed to the persistence of painful TMD (RR = 1.01, 95% CI 1.00–1.03, *P* = 0.047), but it was not related to the transition (RR = 0.98, 95% CI 0.94–1.02, *P* = 0.30). These analyses were adjusted for all covariates. Furthermore, insomnia score was not related to the transition or persistence (RR = 1.00, 95% CI 0.99–1.01, *P* = 0.61). The stratified analysis also showed that the transition (RR = 1.02, 95% CI 0.99–1.01, *P* = 0.16) and persistence (RR = 1.00, 95% CI 0.99–1.01, *P* = 0.88) outcomes were not associated with insomnia score.

### Acute and Chronic Cohort Defined by Dysfunction

At baseline, insomnia (32.50% versus 12.88%, *P* < 0.0001) and EDS (51.57% versus 41.98%, *P* = 0.06) were more common among patients with dysfunction compared to those without (Table 5).

**Table 5.** Baseline profile of the acute and chronic TMD-related pain cohorts defined by dysfunction.

Risk factors and covariates	Category	No dysfunction (GCPS I)	Dysfunction (GCPS II–IV)	*P* value
		132 (29.20%)	320 (70.80%)	
Insomnia, *n* (%)^a^	>14	17 (12.88)	104 (32.50)	<0.0001
	≤14	115 (87.12)	216 (67.50)	
EDS, *n* (%)^b^	>5	55 (41.98)	164 (51.57)	0.06
	≤5	76 (58.02)	154 (48.43)	
Sex, *n* (%)	Female	98 (74.24)	247 (77.19)	0.50
	Male	34 (25.76)	73 (22.81)	
Psychological factors, *n* (%)^c^	≥3	63 (47.73)	212 (66.25)	0.0002
	<3	69 (52.27)	108 (33.75)	
Mean age (95% CI)	Years	39.78 (37.02, 42.53)	42.90 (40.12–44.68)	0.06
Acute to chronic TMD pain defined by pain duration	Acute (*n* = 121)	35 (28.93)	86 (71.07)	0.94
	Chronic (*n* = 331)	97 (29.31)	234 (70.69)	

^a^Insomnia: ≤14 = no, >14 = yes.

^b^EDS: ≤5 = no, >5 = yes.

^c^Psychological factors: ≥3 = yes, <3 = no.

Table 6 shows baseline characteristics of the no dysfunction and dysfunction cohorts that completed the 3-month follow-up. Insomnia (34.33% versus 14.95%, *P* = 0.0002) and EDS (51.50% versus 40.57% *P* = 0.06) were more prevalent in the dysfunction than in the no dysfunction cohort.

**Table 6.** Baseline profile of the no dysfunction and dysfunction cohorts that completed the 3-month follow-up.

Risk factors and covariates	Category	No dysfunction (GCPS I)	Dysfunction (GCPS II–IV)	Total	*P* value
107 (28.53%)	268 (71.47%)	375			
Insomnia, *n* (%)^a^	>14	16 (14.95)	92 (34.33)	108 (28.80)	0.0002
	≤14	91 (85.05)	176 (65.67)	267 (71.20)	
EDS, *n* (%)^b^	>5	43 (40.57)	137 (51.50)	180 (48.39)	0.06
	≤5	60 (59.43)	129 (48.50)	192 (51.61)	
Sex, *n* (%)	Female	80 (74.77)	203 (75.75)	283 (75.47)	0.84
	Male	27 (25.23)	65 (24.25)	92 (24.53)	
Psychological factors, *n* (%)^c^	≥3	55 (51.40)	177 (66.04)	232 (61.87)	0.008
	<3	52 (48.60)	91 (33.96)	143 (38.13)	
Age (years)	Mean	39.47	42.44	42.27	0.11
	Median	38.72	41.00	40.00	
	SD	16.10	16.05	16.35	
Acute to chronic TMD pain defined by pain duration, *n* (%)	Acute	27 (27.27)	72 (72.73)	99 (26.40)	0.75
	Chronic	80 (28.99)	196 (71.01)	276 (73.60)	

^a^Insomnia: ≤14 = no, >14 = yes.

^b^EDS: ≤5 = no, >5 = yes.

^c^Psychological factors: ≥3 = yes, <3 = no.

The number of subjects who completed the 3-month follow-up who presented with or without dysfunction was 268 and 107, respectively. The dysfunction cohort included 90 patients with the persistence of dysfunction (33.58%) compared to 178 without dysfunction (66.42%). From the no dysfunction baseline cohort, eight presented a transition to chronic dysfunction (7.48%) and 99 remained without dysfunction (92.52%).

Table 7 shows the baseline cohort characteristics of patients with or without dysfunction at 3-month follow-up. Insomnia (36.73%, *P* = 0.04) and EDS (59.38%, *P* = 0.01) remained more frequent among patients with dysfunction compared to those without dysfunction.

**Table 7.** Baseline profile of cohorts without or with dysfunction at 3-month follow-up.

Risk factors and covariates	Category	No dysfunction (GCPS I)	Dysfunction (GCPS II–IV)	*P* value
		277 (73.87%)	98 (26.13%)	
Insomnia, *n* (%)^a^	>14	72 (25.99)	36 (36.73)	0.04
	≤14	205 (74.01)	62 (63.27)	
EDS, *n* (%)^b^	>5	123 (44.57)	57 (59.38)	0.01
	≤5	153 (55.43)	39 (40.63)	
Sex, *n* (%)	Female	203 (73.29)	80 (81.63)	0.10
	Male	74 (26.71)	18 (18.37)	
Psychological factors, *n* (%)^c^	≥3	160 (57.76)	72 (73.47)	0.006
	<3	117 (42.24)	26 (26.53)	
Age (years)	Mean	41.63	41.47	0.98
	Median	37.00	42.00	
	SD	17.00	16.06	
Acute to chronic TMD pain defined by pain duration, *n* (%)	Acute	79 (79.80)	20 (20.20)	0.12
	Chronic	198 (71.74)	78 (28.26)	

^a^Insomnia: ≤14 = no, >14 = yes.

^b^EDS: ≤5 = no, >5 = yes.

^c^Psychological factors: ≥3 = yes, <3 = no.

#### The Contribution of Sleep Disorders on the Transition or Persistence of Chronic Painful TMD Defined by Dysfunction

Table 8 shows the findings of the crude and multivariable logistic regression analyses for insomnia and EDS including 375 patients. Insomnia at baseline was associated with the transition or persistence risk based on dysfunction in the crude logistic regression analysis (RR = 1.43, *P* = 0.04). In the multivariable analysis, the RR was weaker and non-significant (RR_ModelII_ = 1.00, *P* = 0.99). To improve the precision of the model, insomnia was not included in model III. In the crude logistic regression analysis, EDS was associated with the increased risk of transition or persistence at a 3-month follow-up (RR = 1.56, *P* = 0.01). However, a borderline association was found in the multivariable analyses (RR = 1.40, *P* = 0.051). In model III, we kept sex and acute and chronic painful TMD in the model even if they were not statistically significant, in contrast to psychological factors and age, because these covariates improved the precision.

**Table 8.** Crude and multivariable logistic regression analyses assessing the contribution of insomnia and EDS on chronic TMD-related pain based on dysfunction at 3-month follow-up using GCPS.

Risk factors and covariates at baseline	Category	Model I RR (95% CI)	*P* value	Model II RR (95% CI)	*P* value	Model III RR (95% CI)	*P* value
Insomnia^a^	≤14	1.0 (reference)	0.04	1.0 (reference)	0.99	Not in the model	
	>14	1.43 (1.02–2.03)		1.00 (0.70–1.43)			
EDS^b^	≤5	1.0 (reference)	0.01	1.0 (reference)	0.16	1.0 (reference)	0.05
	>5	1.56 (1.09–2.22)		1.29 (0.90–1.85)		1.40 (1.00–1.97)	
Acute and chronic TMD-related pain	≤3 months	1.0 (reference)	0.13	1.0 (reference)	0.19	1.0 (reference)	0.15
	>3 months	1.40 (0.91–2.16)		1.32 (0.86–2.02)		1.37 (0.90–2.08)	
Dysfunction^c^	No	1.0 (reference)	<0.0001	1.0 (reference)	<0.0001	1.0 (reference)	<0.0001
	Yes	4.49 (2.25–8.93)		4.09 (2.05–8.19)		4.21 (2.11–8.36)	
Sex	Male	1.0 (reference)	0.11	1.0 (reference)	0.16	1.0 (reference)	0.20
	Female	1.44 (0.92–2.27)		1.37 (0.88–2.16)		1.33 (0.86–2.07)	
Mean age	Years	0.99 (0.98–1.01)	0.93	0.99 (0.98–1.00)	0.37	Not in the model	
Psychological factors^d^	<3	1.0 (reference)	0.008	1.0 (reference)	0.13	Not in the model	
	≥3	1.70 (1.14–2.53)		1.36 (0.91–2.03)			

Model I = crude analysis.

Model II = multivariable model including insomnia and EDS and all covariates: AIC: 398.48, AICC = 398.77, BIC = 429.83.

Model III = multivariable model including EDS and all covariates: AIC: 395.63, AICC = 395.79, BIC = 415.22.

^a^Insomnia: ≤14 = no, >14 = yes.

^b^EDS: ≤5 = no, >5 = yes.

^c^Dysfunction is characterized by a combination of an average of ≥5 over the three pain questions and pain-related disability.

^d^Psychological factors: ≥3 = yes, <3 = no.

#### Secondary Analysis

##### EDS and Insomnia Binary Variables

No interactions were found between insomnia and EDS and dysfunction status (*P* > 0.42). Furthermore, the stratified analysis showed no association between EDS and the transition from acute to chronic pain defined by dysfunction (RR = 2.18, 95% CI 0.56–8.47, *P* = 0.26). A borderline association, however, was found with the persistence of chronic painful TMD (RR = 1.35, 95% CI 0.95–1.91, *P* = 0.09). Insomnia was not associated with persistence risk (RR = 1.12, 95% CI 0.778–1.60, *P* = 0.53). It was not possible to assess the impact of insomnia on the transition risk because in this acute cohort including only the 107 patients, none of the patients with insomnia (*n* = 16) presented a transition.

There were no statistically significant differences between patients who dropped out (*n* = 85) and those who did not (*n* = 441): EDS (*P* = 0.72), acute to chronic (*P* = 0.93), dysfunction (*P* = 0.45), psychological factors (*P* = 0.14), age (*P* = 0.36), sex (*P* = 0.35), and CPI (*P* = 0.81). However, patients who dropped out reported less frequent insomnia (*n* = 13, 16.67%) than those who did not drop out (*n* = 109, 28.34%, *P* = 0.03).

### Inconsistencies in the Reporting of Sensitivity and Specificity of ESS Instrument

Because of this lack of consistency, we opted to assess whether the RR magnitudes and the corresponding 95% CIs were comparable for each group. We also examined the subject count within each group as part of this evaluation. The first crude logistic regression analysis showed that only severe EDS (scores of 16–24) was associated with the transition or persistence of painful TMD: (1) High normal (6–10; *n* = 26/84, RR = 1.07, 95% CI 0.79–1.45, *P* = 0.68), (2) mild excessive (11–12; *n* = 13/17, RR = 1.22, 95% CI 0.96–1.56, *P* = 0.11), (3) moderate excessive (13–15; *n* = 4/16, RR = 0.86, 95% CI 0.62–1.20, *P* = 0.38), (4) severe excessive (16–24; *n* = 6/14, RR = 1.16, 95% CI 1.01–1.35, *P* = 0.04. Comparison = lower normal [0–5]; *n* = 67/128, Akaike information criterion [AIC] = 466.73, corrected AIC [AICC] = 466.90, Bayesian information criterion [BIC] = 486.37). Because the RR magnitude of mild excessive score (11–12) and its 95% CI exhibited a close alignment with severe excessive (16–24), we decided to combine the groups. Our analysis showed that by collapsing scores of 11 to 24, greater EDS remained associated with the transition or persistence of painful TMD (RR = 1.12, 95% CI 1.01–1.35, *P* = 0.04. Comparison = lower normal [0–5]; *n* = 67/128, AIC = 465.87, AICC = 465.94, BIC = 477.65.) Ultimately, given the close alignment between the RR magnitude and the 95% CI of the high normal group (6–10) with that of the mild excessive group (11–24), we concluded that combining these groups would also be appropriate. Furthermore, the AIC, AICC, and BIC values (AIC = 465.69, AICC = 465.72, BIC = 473.54) indicate that this model offers better fit when compared to the previous models. The results are shown in Table 4.

## Discussion

This prospective cohort study demonstrated that EDS was associated with the persistence of painful TMD when chronic painful TMD was defined by duration. Moreover, the RR magnitude and the corresponding 95% CI (RR = 1.35, 95% CI 0.95–1.91, *P* = 0.09) indicated that daytime sleepiness could be considered a predictive factor for the continued presence of a dysfunctional, painful TMD.

The number of participants experiencing EDS in our chronic painful TMD cohort was slightly higher than that found in other studies (Table 1). The OPPERA study showed that patients with TMD were more likely to present OSA when compared to controls. In this OPPERA study, OSA was assessed using three questions from Pittsburgh Sleep Quality Index and four questions from the STOP-BANG.^11^ The OPPERA study had patients with no previous TMD pain or diagnosis and their follow-up group had chronic TMD pain.^11^ Furthermore, the OPPERA prospective study found that individuals with two or more signs/symptoms of OSA had 73% greater incidence of first onset TMD, in relative terms, than those with fewer signs/symptoms, independent of age, gender, race/ethnicity, obesity, smoking history, and autonomic parameters. OSA prevalence as determined by polysomnography among individuals with chronic TMD pain was 28.4%.^10^ Furthermore, the prevalence of TMD was higher among patients with OSA referred for oral appliance therapy.^25^

In the Sanders et al. study,^11^ at baseline, the percentages of very good, fairly good, fairly bad, and very bad sleep quality were 29.2%, 55.5%, and 15.4%, respectively. In our study, at baseline, the percentage of participants experiencing EDS in the acute cohort was 44.7% compared with 49.7% in the chronic cohort (Table 1). In the Sanders et al. study,^11^ Table 1 shows the sleep disorder quality percentages for very good, fairly good, fairly bad, and very bad sleep quality among participants without TMD at baseline. Furthermore, in their case–control analysis (Table 3), the percentages of very good, fairly good, fairly bad, and very bad sleep quality: 28.4%, 53.9%, and 17.7%, respectively. Unfortunately, we did not find a specific frequency of sleep disorders among cases and controls. Therefore, please note that our percentage of 44.7% for the acute cohort and 49.7% for the chronic cohort are close to those presented by the OPPERA study even though they used a different instrument.

We investigated whether the risk would be confounded by the potential confounders such as age, sex, psychological factors (anxiety and depression), CPI, acute and chronic pain status, and dysfunction at baseline. Our baseline findings showed greater dysfunction (GCPS II–IV) in the chronic cohort (70.7%) compared to the acute cohort (71.1%; Table 5). This is consistent with findings from Garofalo et al.^26^ showing a dysfunction prevalence of more than 70% at baseline and 6-month follow-up.

In the Garofalo et al. study,^26^ out of 164 acute patients with painful TMD recruited at baseline, 153 completed the 6-month follow-up (93.3%), and 87 developed chronic painful TMD (56.9%). In Epker et al.’s^27^ study, of 204 patients in the acute cohort, 175 completed the 6-month follow-up (85.8%), and 144 developed chronic TMD (82.3%). In our prospective cohort study, of the acute painful TMD cohort (pain duration ≤3 months since first visit) including 102 patients who completed the 3-month follow-up, half of the patients (*n* = 52) presented a transition to chronic painful TMD, and the other half (*n* = 50) had no pain. Of the chronic painful TMD cohort (pain duration for >3 months since first visit), 75.72% had persistent chronic pain (*n* = 209/276), whereas almost a quarter had no pain at 3-month follow-up (*n* = 67). The difference in percentages might be due to the difference in pain duration and study population.

Insomnia assessed by polysomnography among TMD patients was 36% (*n* = 52) in the chronic painful TMD group.^21^ Barjandi et al.^28^ found that insomnia was prevalent among individuals with myalgia (31.3%) and myofascial pain with a referral (69.1%). Our estimated frequency of insomnia was lower than that study (Tables 1 and 2).

The aim of this study was to assess whether insomnia and EDS were “associated” with the transition from acute to chronic painful TMD as well as its persistence. Therefore, only patients with acute or chronic painful TMD were eligible for participation in this study; patients without painful TMD were not enrolled because it would not be possible to assess transition and persistence outcomes among these cohorts. The prospective cohort study was the best design to achieve our study objectives because it ensured that the risk and potential contributing factors preceded the onset of chronic pain and the persistence of chronic TMD pain.^29^ To decrease the chance of finding a positive association specific to a given hospital because of a referral pattern, patients were recruited from four different dental clinics. To decrease misclassification bias, patients were diagnosed by four different investigators following the same study protocol. To decrease information bias, all questionnaires used were validated and had adequate specificity and sensitivity.^12,20–22^

A limitation of our prospective cohort study relates to different classifications of acute and chronic painful TMD used among researchers. We followed the International Association for the Study of Pain to classify chronic pain (>3 months) and used the GCPS^12^ to classify patients with or without dysfunction to overcome this limitation by avoiding misclassification. Regarding misclassification of potential predictors, EDS with a lower cutoff score might increase the likelihood of false positives due to a lower specificity. This possible baseline misclassification, however, should not be influenced by the outcomes because they were measured at 3-month follow-up; participants who were misclassified as having EDS at baseline were not necessarily misclassified in terms of experiencing the transition or persistence of chronic painful TMD. As a result, the magnitude of the RR would be underestimated rather than overestimated. It is important to note that the impact may not necessarily be in one direction only, and there is uncertainty regarding the specific effect. Another limitation is the sample size of patients with acute painful TMD was not large enough due to the difficulty in recruiting patients with acute TMD even when maximizing our recruitment strategies and efforts at four different clinics. This was the case before and even after COVID-19 closure restrictions were introduced in March 2019. It is possible that a larger sample size in the acute cohort could have strengthened the 80% power of analysis we conservatively estimated. In our study, our dropout rate was 10.2%. Furthermore, the adequacy of power was assessed by analyzing the CIs in addition to considering *P* values, rather than relying solely on *P* values. If the *P* value is slightly greater than 0.05, the CI is skewed toward the right and includes the value of 1, and, while also encompassing clinically meaningful odds ratio (≥2), it can be inferred that this factor is associated with the study outcome. No significant differences were found between patients who dropped out and those who did not. Additionally, the stratified analysis for insomnia had a lower sample size, which contributed to the sample size limitation in our study. Another limitation of our study was not adjusting for unmeasured confounders (e.g., physical conditions and vulnerabilities). Lastly, our team had significant concerns regarding treatment as a potential confounding factor that was not adjusted for in the analysis. This is particularly due to variations in the types of treatments administered (e.g., splints, self-care, pharmacological treatment, physiotherapy, psychological). Several studies^16,30,31^ have investigated the factors influencing persistence but did not include the treatment variable in their analyses.

Our study is the first to demonstrate the potential contribution of EDS to the persistence of chronic painful TMD when chronic pain is defined by duration. The acute cohort was used to study the onset of chronic painful TMD transition, whereas the chronic cohort was used to study the onset persistence of chronic painful TMD. The persistence of chronic painful TMD was defined as the persistence of chronic pain at 3-month follow-up. The magnitude of the RR and *P* value strongly suggest that this factor also contributed to the persistence of chronic pain defined by dysfunction (RR = 1.40, *P* = 0.051). The clinical significance is in the magnitude of the effect. The results of our study are clinically significant because a change in the CI from 1.00 to 1.40 implies a 40% increased risk of transition or persistence, which is of clinical relevance (Table 8). Insomnia, however, was not related to the study outcomes. Though there may be a relationship between sleep disorders and the persistence of painful TMD at 3-month follow-up, we cannot exclude the possibility of a reciprocal relationship where the persistence of painful TMD influences sleep problems and vice versa. Our findings indicate that excessive daytime sleepiness (EDS) screening could be a valuable component of comprehensive TMD assessment, because it may have a predictive role on the transition and persistence of chronic painful TMD. The next research step would be to design a pilot randomized controlled trial to assess specific treatment that could prevent the acute to chronic transition and the persistence of chronic painful TMD. Additionally, evaluating the feasibility of conducting a RCT to measure the efficacy of contemporary current oral appliance therapy for the concurrent management of painful TMD and OSA patients is essential.
